# Statistical fractal analysis in testing the Hypotheses with imprecise data

**DOI:** 10.1016/j.mex.2024.102945

**Published:** 2024-09-12

**Authors:** D. Kalpanapriya, N. Shobana Devi, M. Mubashir Unnissa, Dowlath Fathima

**Affiliations:** a Vellore Institute of Technology, India; b Saudi Electronic University, UAE

**Keywords:** Sampling distribution, Z-test, Statistical fractal, Lacunarity, Testing Hypothesis using Statistical Fractal Method

## Abstract

A new statistical hypothesis testing procedure using fractal analysis, incorporating the concept of lacunarity for interval population parameters when the sample data are real intervals, is introduced. The decision rules for accepting or rejecting the null and alternative hypotheses are provided, along with testing procedures and numerical examples. Additionally, the proposed tests are extended to statistical hypotheses involving fuzzy data samples with lacunarity.

## Graphical abstract

**Figure:**
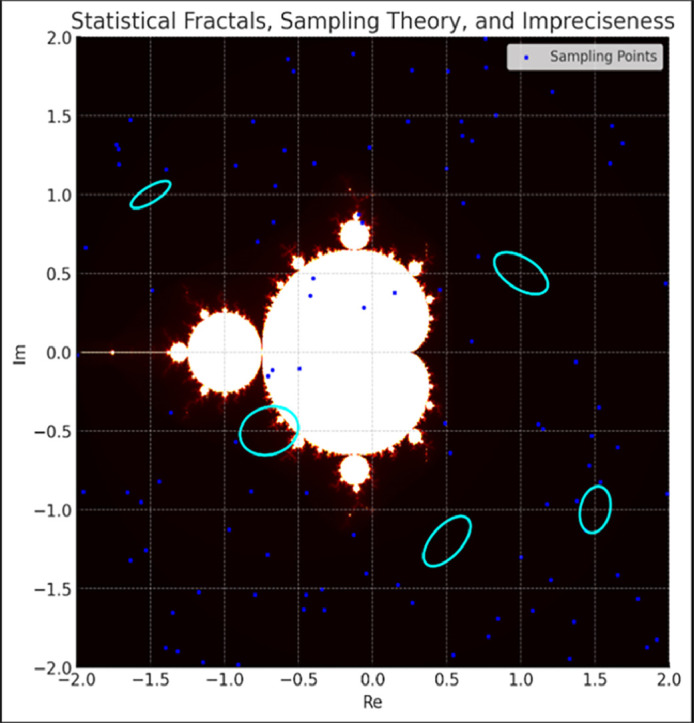

Specifications table

**Table:** 

Subject area:	Mathematics and statistics
More specific subject area:	*Fuzzy Statistics*
Name of your method:	*Testing Hypothesis using Statistical Fractal Method*
Name and reference of original method:	*P.Pandian and D.Kalpanapriya, (2014) “Test of statistical Hypotheses with respect to a Fuzzy set”, Modern applied sciences, Vol.8, pp.1913–1852.*]
Resource availability:	*Not taken any data from any sources].*

## Background

Testing hypotheses are one of the most important areas of statistical analysis. In many situations, the researchers in the field of data analysis are interested in testing a hypothesis about the population parameter. In traditional statistical testing [1], the observations of sample are crisp and a statistical test leads to the binary decision. However, in the real life, the data sometimes cannot be recorded or collected precisely. The statistical hypotheses testing under fuzzy environments have been studied by many authors.

Arnold [2] discussed the fuzzy hypotheses testing with crisp data. The Neyman–Pearson type testing hypotheses were proposed by Casals and Gil [3]. Saade [4] considered the binary hypotheses testing and discussed the fuzzy likelihood functions in the decision-making process. Casals and Gil [5] considered the Bayesian sequential tests for fuzzy parametric hypotheses from fuzzy information. Montenegro et.al. [6] developed two-sample hypothesis tests of means of a fuzzy random variable. Casals and Gill [7] discussed Bayesian sequential test for fuzzy parametric hypotheses from fuzzy information.

The fuzzy tests for hypotheses testing with vague data were proposed by Grzegorzewski [8] and Watanabe and Imaizumi [9]. In the human sciences, Niskanen [10] discussed the applications of soft statistical hypotheses. The statistical hypotheses testing for fuzzy data by proposing the notions of degrees of optimism and pessimism was proposed by Wu [11]. Ahmed K. Elsherif et al. [12] have introduced a new statistical procedure to solve the problem of testing fuzzy hypotheses with fuzzy data based on confidence intervals. Akbari and Rezaei [13] investigated a bootstrap method for inference about the variance based on fuzzy data. Wu [[14], [15]] proposed Analysis of variance for fuzzy data and some approaches to construct fuzzy confidence intervals for the unknown fuzzy parameter.Romer Christof et al. [16] introduced Statistical tests for fuzzy data.Arefi and Taheri [17] developed an approach to test fuzzy hypotheses upon fuzzy test statistic for vague data. Kalpana Priya and Pandian [18] introduced the method which use linguistic data to provide the conclusion about the hypothesis. Zadah [[19], [20]] introduced fuzzy sets and probability measures of fuzzy events.

A Fractal is a type of shape in mathematics which are complex in nature. The whole image looks similar regardless of how much we focus the pattern of every part of the image is called Fractal. In real life we all are surrounded by fractals in different aspects. Fractals are studied by many authors like Kenneth Falconer [21] who introduced Fractal Geometry Mathematical foundation and Application. Plotnick and et.al [22] developed Lacunarity indexes as measure of Landscape texture. Gerald Edgar [23] investigated Measure, Topology and Fractal Geometry. Statistical fractal is a recent development which helps to view the space-time fluctuation pattern in the system dynamically with self-similar structure. Mandelbrot [[24], [25]] introduced the fractal geometry of nature. Siegfried Graf [26] proposed Statistically Self-Similarity Process.

## Method details

### Hypothesis testing for large samples

Statistical hypothesis testing is a technique of statistical inference using sample data which are large or small from a scientific study. These tests are used in determining what outcomes of a study would lead to a rejection of the null hypothesis for a pre-specified level of significance; this can help to decide whether results contain enough information to cast doubt on conventional wisdom, given that conventional wisdom has been used to establish the null hypothesis.

A sample drawn from a population with size more than to 30 is known as large sample. In this section, one population and two population tests for means and variances are listed with decision rules and confidential limits which can be found in Devore [1]. In large sample, sampling distribution approaches a normal distribution and the value of sample statistic is considered the best estimate of the parameter in a population.

### Fuzzy sets and fuzzy numbers

In this section, the basic concepts and important results on fuzzy sets, probability of fuzzy set, fuzzy numbers, arithmetic operations of fuzzy sets and comparison of fuzzy sets and numbers are summarized. These are found in Zadeh [[19], [20]], Chiang and Lin [27] and George J. Klir and Bo Yuan [28].

Let X be a crisp nonempty set and A and B be classical subset of X.

Definition A fuzzy set $\tilde{A}$ defined on X with the membership function $\mu_{\tilde{A}}\left(x\right)$ is defined by

$$\tilde{A}=\left\{\left(x,\mu_{\tilde{A}}\left(x\right)\right):x\in X\text{and}\mu_{\tilde{A}}\left(x\right)\in [0,1]\right\}$$

Definition A real fuzzy number $\tilde{A}=\left(a_{1},a_{2},a_{3}\right)$ is a fuzzy subset from the real line R with the membership function $\mu_{\tilde{A}}\left(x\right)$ satisfying the following conditions.

(i). $\mu {\;}_{\tilde{A}}\;\left(x\right)$ is a continuous mapping from R to the closed interval [0, 1],
(ii). $\mu {\;}_{\tilde{A}}\;\left(x\right)$=0 for every $x\;\in \;\;(-\infty ,a{\;}_{1}\;]$,
(iii). $\mu {\;}_{\tilde{A}}\;\left(x\right)$is strictly increasing and continuous on $\;[a{\;}_{1},\;a{\;}_{2}\;]$,
(iv). $\mu {\;}_{\tilde{A}}\;\left(x\right)$= 1 for every $a\;\;=a{\;}_{2}\;$,
(v). $\mu {\;}_{\tilde{A}}\;\left(x\right)$is strictly decreasing and continuous on $\left[\;a{\;}_{2},\;a{\;}_{3}\;\;\right]$ and
(vi). $\mu {\;}_{\tilde{A}}\;\left(x\right)$=0for every $x\in \;[a{\;}_{3},+\infty )\;$.

Definition A fuzzy number $\tilde{A}$ is a triangular fuzzy number denoted by $\left(a_{1},a_{2},a_{3}\right)$ where $a_{1},a_{2}\text{and}a_{3}$ are real numbers and its member ship function $\mu {\;}_{\tilde{A}}\;\left(x\right)$ is given below:

$$\mu_{\tilde{A}}\left(x\right)=\{\begin{array}{c} \left(x-a_{1}\right)/\left(a_{2}-a_{1}\right)\\ \left(a_{3}-x\right)/\left(a_{3}-a_{2}\right)\\ 0 \end{array}\begin{array}{c} \;\;\;\text{for}\;\;a_{1}\leq x\leq a_{2}\\ \;\;\;\text{for}\;\;a_{2}\leq x\leq a_{3}\\ \text{otherwise} \end{array}.$$

The graph of $\mu {\;}_{\tilde{A}}\;\left(x\right)$ is given below:

**Figure:**
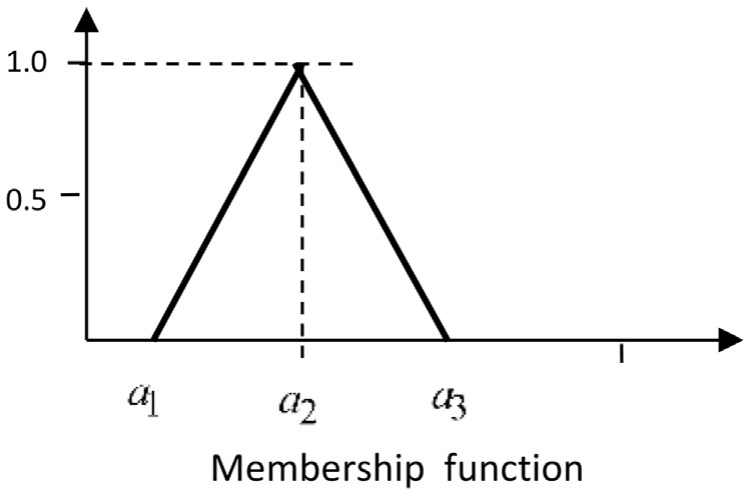

Definition Let $\tilde{A}$ and $\tilde{B}$ be two fuzzy sets defined on X such that $\tilde{A}=\left\{\langle x,\mu_{\tilde{A}}\left(x\right)\rangle :x\in X\right\}$; $\tilde{B}=\left\{\langle x,\mu_{\tilde{B}}\left(x\right)\rangle :x\in X\right\}$. Then,

(i). $\tilde{A}\subseteq \tilde{B}\;iff\;\mu_{\tilde{A}}\left(x\right)\leq \mu_{\tilde{B}}\left(x\right)\;$for each x∈X.
(ii). $\tilde{A}⊇\tilde{B}\;iff\;\mu_{\tilde{A}}\left(x\right)\geq \mu_{\tilde{B}}\left(x\right)\;$for each x∈X.
(iii). $\tilde{A}=\tilde{B}\;iff\;\mu_{\tilde{A}}\left(x\right)=\mu_{\tilde{B}}\left(x\right)\;$for each x∈X.
(iv). $\tilde{A}\cup \tilde{B}=\{\left(x,\max.\{\;\mu_{\tilde{A}}\left(x\right),\mu_{\tilde{B}}\left(x\right)\}\;\right),x\in X\}$ and
(v). $\tilde{A}\cap \tilde{B}=\{\left(x,\min.\{\;\mu_{\tilde{A}}\left(x\right),\mu_{\tilde{B}}\left(x\right)\}\;\right),x\in X\}$

Definition Let $\tilde{A}$ be a fuzzy set defined on X with MF,$\mu_{\tilde{A}}\left(x\right)$. Then, the probability of a fuzzy set is given by

(1) $$P\left(\tilde{A}\right)=\int_{x}\mu_{\tilde{A}}\left(x\right)dP=E\left(\mu_{\tilde{A}}\left(x\right)\right)$$

where *P* is the probability measure over *X*. From (1), we can say that if the probability measure of *X* is known, the probability of the occurrence of the fuzzy event $\tilde{A}$ is the mean of $\mu_{\tilde{A}}\left(x\right)$.

Definition Let $\left\{x_{1},x_{2},...,x_{n}\right\}$ be a random sample of size *n* from a crisp set *X* with the membership grades (MGs) of a fuzzy set $\tilde{A}$ where $\tilde{A}=\left\{\left(x,\mu_{\tilde{A}}\left(x\right)\right)/x\in X\right\}$. Then, the sample mean of the MF of the fuzzy set $\tilde{A}$ defined on *X* or the average MGs of fuzzy set $\tilde{A}$ over the random sample denoted by ${\bar{\mu }}_{\tilde{A}}\left(x\right)$ is given by

(2) $${\bar{\mu }}_{\tilde{A}}\left(x\right)=\frac{1}{n}\left(\sum_{i=1}^{n}\mu_{\tilde{A}}\left(x_{i}\right)\right)$$

Definition Let $\left\{x_{1},x_{2},...,x_{n}\right\}$ be a random sample of size *n* from a crisp set *X* with the MGs of a fuzzy set $\tilde{A}$ where $\tilde{A}=\left\{\left(x,\mu_{\tilde{A}}\left(x\right)\right)/x\in X\right\}$. Then, the sample variance of the MF of the fuzzy set $\tilde{A}$ defined on *X* or the variance of the MGs of fuzzy set $\tilde{A}$ over the random sample denoted by $S_{\tilde{A}}^{2}\left(x\right)$ is given by and $S_{\tilde{A}}\left(x\right)$ is the sample standard deviation of the MF of the fuzzy set $\tilde{A}$ defined on *X.*

(3) $$S_{\tilde{A}}^{2}\left(x\right)=\frac{1}{n=1}\left(\sum_{i=1}^{n}{\left(\mu_{\tilde{A}}\left(x_{i}\right)-{\bar{\mu }}_{\tilde{A}}\left(x\right)\right)}^{2}\right)$$

### Lacunarity index for interval parameters

Lacunarity, from the Latin lacuna, meaning "gap" or "lake", is a specialized term in geometry referring to a measure of how patterns, especially fractals, fill space, where patterns having more or larger gaps generally have higher lacunarity. The lacunarity using interval parameters (Mean and Variance) is given by

(4) $$\left[L^{l}\right]=1+\left[\left[v^{l}\right]\;/\;{\left[m^{l}\right]}^{2}\right]\;\text{and}\;\left[L^{u}\right]=1+\left[\left[v^{u}\right]\;/\;{\left[m^{u}\right]}^{2}\right]$$

Theorem The components of totally disconnected space are its points. (Jayavelu et.al. [29])

Proof It is sufficient to prove that any subspace of X with more than one point is disconnected. Let Y be a subspace of X with more than one point. Since X is totally disconnected if x, y ∈ Y if x ∉ y then x, y ∈ X and there exist a disconnection of x i.e., *X* = *A*∪B, A∩*B* = ∅, A, B are open sets in X where x ∈ A, y ∈ B. Then *Y* = (A∩Y) ∪ (B∩Y) is a disconnection of Y. That is Y is not connected.

Hence any subspace of X having more than one point is disconnected. Hence the component of totally disconnected space are its point [13,17].

### Hypothesis testing for interval population parameters with large samples

In this section, we propose the following imprecise hypotheses testing for large samples:

Imprecise hypothesis testing for single population interval mean and imprecise hypothesis testing for difference between interval mean values of two populations using Statistical fractal analysis with the degree point lacunarity is introduced.

### Hypothesis testing for single population interval mean with the degree point lacunarity

Let $\left\{[x_{i},y_{i}],i=1,2,...,n\right\}$ be a random large sample such that $\left\{x_{i},i=1,2,...,n\right\}$ is a random sample from a normal population and $\left\{y_{i},i=1,2,...,n\right\}$ is another random sample from another normal population and the population mean of the sample be [a,b].

Now, we are going to test the null hypothesis that the population mean of the given sample [a,b] is equal to a specified interval $\left[a_{\circ },b_{\circ }\right]$,that is, $a=a_{\circ }$ and $b=b{}_{\circ }$.

**Testing Hypothesis:**

Null Hypothesis $H_{0}$: $\left[a,b\right]=\left[a_{\circ },b_{\circ }\right]$

Alternative Hypothesis $H_{A}$

(i). $\left[a,b\right]\neq \left[a_{\circ },b_{\circ }\right]$
(ii). $\left[a,b\right]≻\left[a_{\circ },b_{\circ }\right]$
(iii). $\left[a,b\right]≺\left[a_{\circ },b_{\circ }\right]$

Consider the random sample of lower value of given interval data, $X_{l}=\left\{x_{i},i=1,2,...,n\right\}$ and the another random sample of upper value of the given interval data, $X_{u}=\left\{y_{i},i=1,2,...,n\right\}$.

Now, the sample mean of $X_{l}$ and $X_{u}$ are $\bar{x}$ and $\bar{y}$ respectively and the sample S.D. of $X_{l}$ and $X_{u}$ are $\sigma_{x}$ and $\sigma_{y}$respectively.

Test statistics:

(5) $$Z_{l}=\frac{\left(\bar{x}-\eta \right)\sqrt{n}}{\sigma_{x}}\;\text{and}\;Z_{u}=\frac{\left(\bar{y}-\mu \right)\sqrt{n}}{\sigma_{y}}$$

The acceptance or rejection of Null hypothesis is based on the theorem about the samples of X and Y are totally disconnected or not.

Now, the 100(1−α)% confidence limits for the population mean [η,μ] corresponding to the given sample are given below:

(6) $$\left[\bar{x}-Z_{\alpha /2}\left(\frac{\sigma_{x}}{\sqrt{n}}\right),\bar{y}-Z_{\alpha /2}\left(\frac{\sigma_{y}}{\sqrt{n}}\right)\right]<[a,b]<\left[\bar{x}+Z_{\alpha /2}\left(\frac{\sigma_{x}}{\sqrt{n}}\right),\bar{y}+Z_{\alpha /2}\left(\frac{\sigma_{y}}{\sqrt{n}}\right)\right]$$

Now, the above said test procedure can be illustrated using the following imprecise data with the example.

Example Suppose that the standard deviation of the heights of university male students is between $2^{\prime \prime }$ and $5^{\prime \prime }$.One hundred male students of a university are measured and their range of mean height is found to be $\left[65^{\prime \prime },68^{\prime \prime }\right]$. Determine if this mean height represents a significant difference from the population interval mean of $\left[\;64^{\prime \prime },71^{\prime \prime }\right]$ using the degree of Lacunarity.

We are now going to test the null hypothesis $H_{0}$:$\left[a\right]=\left[a_{\circ }\right]$ against the alternative hypothesis $H_{A}$: $\left[a\right]\neq \left[a_{\circ }\right]$.

The size of the sample, n=100 and the population mean is $\left[\mu_{\circ }\right]=\left[\;64^{\prime \prime },71^{\prime \prime }\right]$ with known sample S.D $\left[2^{\prime \prime },\;5^{\prime \prime }\right]$ . Let the level of significance be 5 %.

Now, the mean value of the lower and upper interval values are ${\bar{x}}^{l}=\;65^{\prime \prime }$ and ${\bar{x}}^{u}=\;68^{\prime \prime }$ respectively and the S.D. of the lower and upper interval values are $\sigma^{l}=2^{\prime \prime }$ and $\sigma^{u}=5^{\prime \prime }$ respectively.

Now, the table value of Z at 5 % level, T=1.96 .

Now the lower value of $\left[a_{\circ }\right],\;{a_{\circ }}^{l}=64^{\prime \prime }$ and the upper value of $\left[a_{\circ }\right],\;\;{a_{\circ }}^{u}=71^{\prime \prime }.$

Now, $Z^{l}=5$ and $Z^{u}=6$

Decision table about NH, $H_{0}$

From the calculated mean and variance. Lacunarity is obtained by

$[L^{l}]=1.0009$ and $[L^{u}]=1.005$

Therefore [L]=[1.0009,1.005]

The degree of point in Lacunarity explains that there is no significant difference between the sample means and population means.

### Hypothesis testing for difference between interval mean values of two populations with the degree point lacunarity

Let $\left\{[x_{i},y_{i}],i=1,2,...,m\right\}$ be a random small sample (X- sample) and $\left\{[u_{j},v_{j}],i=1,2,...,n\right\}$ be another random small sample (Y-sample) such that $\left\{[x_{i},y_{i}],i=1,2,...,m\right\}$ is a random sample from a normal population with mean $\left[a_{1},b_{1}\right]$ and $\left\{[u_{j},v_{j}],i=1,2,...,n\right\}$ is another random sample from another normal population with mean $\left[a_{2},b_{2}\right]$ .

Now, we are going to test the null hypothesis that the means of the populations of the given samples are equal, that is, $\left[a_{1},b_{1}\right]=\left[a_{2},b_{2}\right]$ this implies that, $a_{1}=a_{2}$ and$b_{1}=b{}_{2}$.

**Testing Hypothesis:**

Null Hypothesis ($H_{\circ }$):$\left[a_{1},b_{1}\right]=\left[a_{2},b_{2}\right]$ that is $a_{1}=a_{2}$ and $b_{1}=b{}_{2}$.

Alternative Hypothesis ($H_{A}$)

(i). $\left[a_{1},b_{1}\right]\neq \left[a_{2},b_{2}\right]$ that is, $a_{1}\neq a_{2}$ or $b_{1}\neq b{}_{2}$.
(ii). $\left[a_{1},b_{1}\right]>\left[a_{2},b_{2}\right]$ that is, $a_{1}>a_{2}$ and $b_{1}>b{}_{2}$.
(iii) $\left[a_{1},b_{1}\right]<\left[a_{2},b_{2}\right]$ that is, $a_{1}<a_{2}$ and $b_{1}<b{}_{2}.$

Consider the following random sample consisting of the lower values of X- sample and Y-sample:

**Table:** 

$X^{l}$ (lower values of X-sample)	$x_{i},\;i=1,2...m$
$Y^{l}$ (lower values of Y-sample)	$u_{j},j=1,2...n$

Now, the sample means of $X^{l}$ and $Y^{l}$ are ${\bar{x}}_{l}$ and ${\bar{y}}_{l}$ respectively and the sample S.Ds of $X^{l}$ and $Y^{l}$are $\sigma_{xl}$ and $\sigma_{yl}$ respectively.

Consider the following random sample consisting of the upper values of X- sample and Y-sample:

**Table:** 

$X^{u}$ (upper values of X-sample)	$y_{i},\;i=1,2...m$
$Y^{u}$ (upper values of Y-sample)	$v_{j},j=1,2...n$

Now, the sample means of $X^{u}$ and $Y^{u}$ are ${\bar{x}}_{u}$ and ${\bar{y}}_{u}$ respectively and the sample S.Ds of $X^{u}$ and $Y^{u}$ are $\sigma_{xu}$ and $\sigma_{yu}$respectively.

If the population standard deviations are assumed to be equal, we use the following test statistics .

(7) $$Z_{l}=\frac{{\bar{x}}_{l}-{\bar{y}}_{l}}{\sigma_{l}\sqrt{\frac{1}{m}+\frac{1}{n}}}\;\text{and}\;Z_{u}=\frac{{\bar{x}}_{u}-{\bar{y}}_{u}}{\sigma_{u}\sqrt{\frac{1}{m}+\frac{1}{n}}}$$

where

(8) $$\sigma_{l}=\sqrt{\frac{\left(m-1\right)\sigma_{x_{l}}^{2}+\left(n-1\right)\sigma_{y_{l}}^{2}}{m+n-2}}\;\text{and}\;\sigma_{u}=\sqrt{\frac{\left(m-1\right)\sigma_{x_{u}}^{2}+\left(n-1\right)\sigma_{y_{u}}^{2}}{m+n-2}}$$

The acceptance or rejection of Null hypothesis is based on the theorem about the samples of X and Y are totally disconnected or not.

Now, the 100(1−α)% confidence limits for the difference of lower limit and upper limit of the population means $\left[a_{1},b_{1}\right]$ and $\left[a_{2},b_{2}\right]$corresponding to the given samples are given below:

(9) $$\begin{array}{c} ({\bar{x}}_{l}-{\bar{y}}_{l})-Z_{\alpha /2}\left(\sigma_{Y}\sqrt{\frac{1}{m}+\frac{1}{n}}\right)<a_{1}-a_{2}<({\bar{x}}_{l}-{\bar{y}}_{l})+Z_{\alpha /2}\left(\sigma_{Y}\sqrt{\frac{1}{m}+\frac{1}{n}}\right) \end{array}$$

and

(10) $$\begin{array}{c} ({\bar{x}}_{u}-{\bar{y}}_{u})-Z_{\alpha /2}\left(\sigma_{Y}\sqrt{\frac{1}{m}+\frac{1}{n}}\right)<b_{1}-b_{2}<({\bar{x}}_{u}-{\bar{y}}_{u})+Z_{\alpha /2}\left(\sigma_{Y}\sqrt{\frac{1}{m}+\frac{1}{n}}\right) \end{array}$$

The test procedure can be illustrated using the following example having imprecise data.

Example In a survey of shopping habits 400 women shoppers are chosen at random in supermarket ‘A’ located in a certain part of the Vellore city, their minimum and maximum average weekly food expenditure is [Rs.235,Rs.255] with standard deviation [Rs.36,Rs.42] . For 400 women shoppers are chosen at random in supermarket ‘B’ located in another part of the Vellore city, their minimum and maximum average weekly food expenditure is [Rs.216,Rs.228] with standard deviation [Rs.48,Rs.57]. Test at 1 % level of significance whether the minimum and maximum average weekly food expenditures of the populations of the shoppers are equal using the degree of Lacunarity.

Therefore we have: $m=n=400,\;{\bar{x}}_{l}=235,\;{\bar{y}}_{l}=216,\;{\bar{x}}_{u}=255,\;{\bar{y}}_{u}=228,\;\sigma_{x}^{l}=36,\;\sigma_{y}^{l}=42$ and $\sigma_{x}^{u}=48,\;\sigma_{y}^{u}=57.$

Null hypothesis, $\left[H_{0}\right]$: The average weekly food expenditures of the populations are equal

And the alternative hypothesis, $[H_{a}]:$ The average weekly food expenditures of the populations are not equal.

This implies that $\left[H_{0}\right]:\;a_{1}=a_{2}$ and $\;b_{1}=b_{2}$; $\left[H_{A}\right]:\;a_{1}\neq a_{2}$ or $\;b_{1}\neq b_{2}$

Test statistics:

$Z_{l}=6.869$ and $Z_{U}=7.2466$

Decision table about NH, $H_{0}$

From the calculated mean and variance. Lacunarity is obtained by

$[L^{l}]=1.0075$ and $[L^{u}]=1.012$

Therefore [L]=[1.0075,1.012]

The degree of point in Lacunarity explains that there is no significant difference between the minimum and maximum average weekly food expenditures of the populations of the shoppers are equal.

## Method validation

Tests of statistical hypotheses for large samples for imprecise data using statistical fractal analysis were discussed. This study is totally different from conventional statistical hypothesis testing. In the proposed tests of hypotheses, the differences of means with respect to fuzzy sets satisfy the self- similarity of fractals by statistical fractals using Lacunarity. Further, we provide the rules for decision taken about the hypotheses. The proposed tests of hypotheses can help decision makers in linguistic hypotheses tests related issues of real-life problems by aiding them in the decision-making process and providing an appropriate decision rules in an acceptable manner. Nevertheless, there will be self-similarity [26] since both samples share the same shape according to statistical fractals. Thus, the fractal property is upheld. According to the theorem, X and Y are entirely disconnected. The interval lacunarity measure accurately captures the degree of disconnection between points in a set.

## Limitations

Not applicable.

## Ethics statements

Work not includes the following: Human subjects, Animal experiments and social media.

## CRediT author statement

Kalpanapriya: Conceptualization, Methodology, Validation, Writing – review & editing. Shobanadevi: Methodology, Resources. Mubashir unnissa: Investigation. Dowlath fathima: review & editing.

## Declaration of competing interest

The authors declare that they have no known competing financial interests or personal relationships that could have appeared to influence the work reported in this paper.

## Data Availability

No data was used for the research described in the article.
